# Dominance of Sulfur-Oxidizing Bacteria, *Thiomicrorhabdus*, in the Waters Affected by a Shallow-Sea Hydrothermal Plume

**DOI:** 10.3390/biology14010028

**Published:** 2025-01-01

**Authors:** Chih-Ching Chung, Gwo-Ching Gong, Hsiao-Chun Tseng, Wen-Chen Chou, Chuan-Hsin Ho

**Affiliations:** 1 Institute of Marine Environment and Ecology, National Taiwan Ocean University, 2 Pei-Ning Road, Keelung 202, Taiwan; 2 Center of Excellence for the Oceans, National Taiwan Ocean University, 2 Pei-Ning Road, Keelung 202, Taiwan; 3 Department of Science Education, National Taipei University of Education, 134 Section 2, Heping East Road, Taipei City 106, Taiwan

**Keywords:** hydrothermal vent, chemolithotroph, picoplankton

## Abstract

The shallow-sea hydrothermal vent at Guishan Islet, located off the coast of Taiwan, serves as a remarkable natural site for studying microbial ecology in extreme environments. In April 2019, we investigated the composition of prokaryotic picoplankton communities, their gene expression profiles, and the dissolved inorganic carbon uptake efficiency. Our results revealed that the chemolithotrophs *Thiomicrorhabdus* spp. contributed to the majority of primary production in the waters affected by the hydrothermal vent plume. The metatranscriptomic analysis aligned with the primary productivity measurements, indicating the significant gene upregulations associated with carboxysome-mediated carbon fixation in *Thiomicrorhabdus*. *Synechococcus* and *Prochlorococcus* served as the prokaryotic photoautotrophs for primary productivity in the waters with lower influence from hydrothermal vent emissions. *Thiomicrorhabdus* and picocyanobacteria jointly provided organic carbon for sustaining the shallow-sea hydrothermal vent ecosystem. In addition to the carbon fixation, the upregulation of genes involved in the SOX (sulfur-oxidizing) pathway, and the dissimilatory sulfate reduction indicated that energy generation and detoxification co-occurred in *Thiomicrorhabdus*. This study improved our understanding of the impacts of shallow-sea hydrothermal vents on the operation of marine ecosystems and biogeochemical cycles.

## Simple Summary

An investigation into the prokaryotic picoplankton ecology and their assimilation ability in dissolved inorganic carbon was conducted in the shallow-sea hydrothermal vent at Guishan Islet, located off the coast of Taiwan in April 2019. We found that the thiosulfate-oxidizing bacteria, *Thiomicrorhabdus*, were the dominant primary producer in the waters mixing with the hydrothermal vent plume. In waters with less influence from hydrothermal vent emissions, picocyanobacteria (i.e., *Synechococcus* and *Prochlorococcus*) acted as the primary prokaryotic photoautotrophs. The combined contributions of *Thiomicrorhabdus* and picocyanobacteria were vital in sustaining primary production and supporting the shallow-sea hydrothermal ecosystem.

## 1. Introduction

Hydrothermal vents are often found on the sea floors where the tectonic plates meet or where volcanic activity frequently occurs. The plume from a vent is seawater heated by hot magma and returns to the sea floor. It usually has a higher temperature and is rich in methane, sulfides, metals, and minerals [1]. Because hydrothermal vents with unique hydrological characteristics are usually located in the deep sea where sunlight does not reach, most microorganisms here are chemolithotrophs, using energy from redox reactions to turn dissolved inorganic carbon (DIC) into organic compounds. The organic carbon synthesized by these chemolithotrophic bacteria is mainly an energy source used to sustain the ecosystem of the deep-sea hydrothermal vents [2]. Besides the deep-sea hydrothermal vents, some hydrothermal vents are found on the sea floor with a depth of or less than 200 m. In shallow-sea hydrothermal vent ecosystems, sunlight can penetrate the entire water layer, and hydrothermal plumes can also affect the surrounding water body. Therefore, chemolithotrophic and photoautotrophic nutritional modes can coexist. The emergence of two autotrophic modes provides various primary production sources that fuel the biogeochemical cycle and contribute to a distinct community structure in the shallow-sea hydrothermal vent ecosystem [3,4,5,6,7,8].

The microbial community compositions in the shallow-sea hydrothermal vents, like in the deep-sea hydrothermal fields, have also been known to be dominated by chemolithotrophic bacteria. In the shallow-sea hydrothermal plumes rich in hydrogen sulfide or thiosulfate, sulfur-oxidizing autotrophic bacteria dominate due to their ability to obtain energy through sulfide oxidation, facilitating the conversion of dissolved inorganic carbon (DIC) into organic carbon [9,10,11]. Photoautotrophs should be another essential primary producer in the shallow-sea hydrothermal areas attributed to sunlight penetration. Research comparing the contributions of primary production provided by chemolithotrophs and photoautotrophs in shallow-sea hydrothermal regions is limited. According to the microscopic observation or the 16S ribosomal RNA gene diversity, picocyanobacteria were suggested to account for less than 10% of total bacterial abundance. It has been suggested that chemolithotrophs are the principal primary producers in the shallow-sea hydrothermal fields [5,6,9,11]. However, such inference was only supported by microbiological observations. The *in situ* measurement of primary production still needs to be carried out. Using the carbon isotope labeling method, Sorokin et al. (1998) found that the efficiency of DIC fixation in the water-covered active shallow-sea volcanoes was 10 to 30 times greater than the adjacent oceanic water [6]. Similar phenomena were also reported by Lin et al. (2021) [4]. These studies suggested that the chemolithotrophs were responsible for DIC fixation in the shallow-sea hydrothermal vent area.

Guishan Islet is located offshore of Northeast Taiwan, where the Kuroshio current flows through. Vigorous hydrothermal activity occurs in the seafloor within 30 m of depth in the east of this Islet. The gas discharged from the vent, which comprises carbon dioxide, nitrogen, hydrogen sulfide, and methane, is immediately mixed with the surrounding seawater, attributed to the strong flow of Kuroshio current [12,13]. Previous studies have indicated that chemolithotrophic bacteria and picocyanobacteria co-occurred in the hydrothermal plume and surrounding waters [3,4,11]. Although it can be inferred that the primary production from photosynthesis and chemolithotrophs should cooperatively drive the operation of the food chain in the hydrothermal vent of Guishan Islet, there is still a lack of in situ measures to elucidate the community composition of planktonic microorganisms and their efficiency of carbon fixation simultaneously. Moreover, previous studies have primarily focused on areas near hydrothermal vents. Limited research has examined how the interaction between hydrothermal vent plumes and surrounding seawater affects microbial ecology. Especially in the highly oligotrophic photic layer water bodies of the subtropical Kuroshio, any input of nutrients significantly impacts primary productivity and the maintenance of the food chain. In light of this, we assessed the carbon fixation efficiency and the microbial community composition in the waters surrounding Guishan Islet. In addition, metatranscriptomic analysis was performed to profile microbial gene expression, uncovering potential molecular regulatory mechanisms involved in chemolithotrophic carbon fixation and sulfur metabolism within this ecosystem. Simultaneous analyses of hydrographic characteristics, primary productivity, microbial community composition, and gene expression were conducted to assess the impact of this shallow-sea hydrothermal vent on marine ecology and biogeochemical cycles. Our research will enhance our understanding of microbial community composition and its relationship with biogeochemical cycles in shallow-sea hydrothermal ecosystems.

## 2. Materials and Methods

### 2.1. Sample Collection

**Figure 1:**
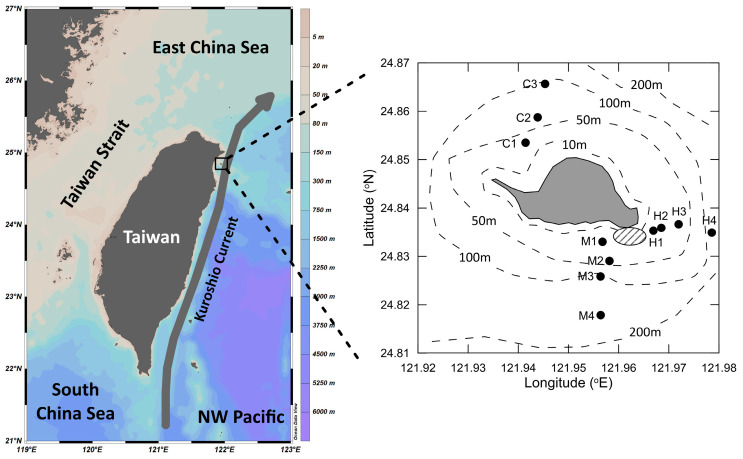
The map of the Guishan Islet and the sampling locations of the stations. The area marked with dashes in (**Right**) is the location of the shallow-sea hydrothermal vent. Stations H1 and M1 are near the shallow-sea hydrothermal vent. The waters from Stations H2 to H4 and M2 to M4 are mixed with the hydrothermal vent plume and Kuroshio seawater. The waters from Stations C1 to C3 are seldom affected by hydrothermal vent plume. The extent of mixing varies according to the distance from the hydrothermal vent.

The R/V Ocean Researcher II voyage was conducted in the sea around the Guishan Islet from 15 to 17 April 2019 (Figure 1). Temperature and salinity were measured by a conductivity/temperature/depth recorder (CTD) (SBE911 plus, Sea-Bird Electronics, Bellevue, WA, USA). Dissolved oxygen saturation (DOS) and particle transmission (TM) in different water layers were measured with a DO sensor (SBE 43, Sea-Bird Electronics, Bellevue, WA, USA) and a transmissometer (Alpha Tracka II, Chelsea Technologies Group, Yateley, Hampshire, UK). These probes were equipped on the CTD rosette. To determine hydrography, inorganic carbon fixation rates, and assemblage composition and gene expression dynamics of bacteria, the water samples were collected using Teflon-coated 20 L Niskin bottles (General Oceanics, Miami, FL, USA) mounted on the CTD rosette sampler from the surface layer to the depth about 5 m from the seafloor. If the water depth exceeded 100 m, sampling was limited to the top 100 m. For the detailed depth of each sample collection, please refer to Supplementary Table S1.

### 2.2. Determination of Hydrographic Features

The sample for the measurements of dissolved inorganic nutrients was placed in a polypropylene bottle and immediately frozen with liquid nitrogen. NH_4_ was measured using the indophenol blue method [14]. NO_2_ and NO_3_ were analyzed using the pink azo dye method with a self-designed flow injection analyzer [15,16]. PO_4_ and SiO_4_ were measured by the molybdenum blue method [15,17]. The detection limits of NH_4_, NO_2_, NO_3_, PO_4_, and SiO_4_ are 0.06, 0.01, 0.3, 0.03, and 0.2 μM, respectively.

To stop biological activity, samples for the determination of CH_4_ and pH were added to saturated HgCl_2_ solution at a final concentration of 0.05% (*v*/*v*). The CH_4_ was measured with the head-space equilibration technique using a gas chromatograph (Agilent 7890, Agilent Technologies, Santa Clara, CA, USA) fitted with a flame ionization detector. The primary standard was 1.16 ppmV CH_4_ (MESA Specialty Gas, Santa Ana, CA, USA). The precision of repeated analysis of water samples for CH_4_ measurement was about ±3% [18,19]. The pH value was spectrophotometrically (UV-1800, Shimadzu, Kyoto, Japan) measured using unpurified meta-cresol purple (Tokyo Chemical Industry, Tokyo, Japan). The pH value was determined based on the total hydrogen ion concentration scale with a precision of 0.005 [20,21].

Samples for chlorophyll a (chl. a) measurements were collected by filtering 500 mL of seawater through a 47 mm diameter GF/F filter (Whatman, Maidstone, Kent, UK) and were stored at −20 °C until analysis. Chl *a* retained on the filter was extracted with 90% acetone and then measured with a fluorometer (Model 10-AU-005, Turner Design, San Jose, CA, USA) [15,22,23].

### 2.3. Determination of Dissolved Inorganic Carbon (DIC) Fixation Efficiency

The water sample for the measurement of DIC fixation efficiency was filtered through a 200 μm mesh, and the filtrate was filled into a 10 L polycarbonate bottle (Nalgene, Rochester, NY, USA). The subsamples were injected with 10 μCi NaH^14^CO_3_. They were incubated in the dark and under the light intensity at 2200 μE m^−^^2^ s^−^^1^ (1000 W submersible halo-gen quartz lamp) in the seawater-cooled incubator for 2 h. Subsequently, the cells in the sample were collected by the GF/F filter (Whatman, Maidstone, Kent, UK) under a gentle vacuum (<100 mmHg). The filters were placed in the scintillation vials and stored in the dark until analysis. The residual NaH^14^CO_3_ on the filter was removed by adding 0.1 N HCl to volatilize overnight. After the addition of the scintillation cocktail (Ultima Gold, PerkinElmer, Shelton, CT, USA), the radioactivity of ^14^C-labeled particulate organic materials on the filters was counted in a liquid scintillation analyzer (Tri-Card 2800TR, PerkinElmer, Shelton, CT, USA) [24].

### 2.4. Enumeration of Picoplankton

Samples for picoplankton enumeration were fixed in seawater-buffered paraformaldehyde at a final concentration of 0.2% (*w*/*v*). The samples were frozen in liquid nitrogen and preserved at −80 °C until analysis. A flow cytometry (FACSAria, Becton-Dickinson, Franklin Lakes, NJ, USA) was used to distinguish different picoplankton populations. For *Synechococcus*, *Prochlorococcus*, and eukaryotic picophytoplankton, their abundances were identified and enumerated based on the cell size (forward- and side-scattering) and their autofluorescence in the range of orange (from phytoerythrin, 575 ± 15 nm) and red (from chlorophyll, ≥670 nm). Bacteria stained with the SYBR-Green I dye (Molecular Probes, Eugene, OR, USA) were counted in a separate subsample. In addition, a known number of fluorescent beads (TruCOUNT Tubes, Becton-Dickson, Franklin Lakes, NJ, USA) were calculated to convert the original cell abundance in the sample [25,26].

### 2.5. Environment DNA Isolation

The two-liter water sample was filtered through a 5-µm mesh nylon net to remove larger plankton. The small planktonic cells in the filtrate were simultaneously harvested on six 0.2-µm pore-size polycarbonate membranes (diameter = 45 mm) (Nucleopore, Whatman, Maidstone, Kent, UK) under gentle vacuum (<100 mmHg). The membranes were pooled together in one cryovial and immediately frozen in liquid nitrogen until DNA isolation. The method for DNA isolation was described by Chung et al. (2015) [25]. In brief, the cells were disrupted by lysozyme (Roche, Grenzacherstrasse, Switzerland) and proteinase K treatments (Roche, Grenzacherstrasse, Switzerland). After purification by hexadecyltrimethylammonium bromide and phenol/chloroform/isoamyl alcohol (25/24/1, *v*/*v*/*v*), the DNA pellet was precipitated using isopropanol and resuspended in the Tris-EDTA buffer (pH 8.0). The concentration and purity of DNA were determined by spectrophotometry (NanoDrop, Thermo Fisher Scientific, Wilmington, DE, USA) at wavelengths of 230, 260, 280, and 320 nm.

### 2.6. Environment Total RNA Isolation

The five-liter water sample was filtered through a 5-µm mesh nylon net to remove larger plankton. The sample collection was the same as the DNA extraction described above, except that the entire process must be completed quickly (usually within 20 min) to avoid changes in mRNA composition. The filter membranes containing small planktonic cells were immediately immersed in a lysis buffer (Qiagen, Hilden, Germany) and stored in liquid nitrogen until processing. Before the RNA isolation, the membranes were ground by a bead beater (Biospec Products, Bartlesville, OK, USA) with an appropriate amount of glass beads (diameter = 0.1 mm) to disrupt the cells completely. The total RNA in the lysis buffer was isolated by the RNeasy Plant kit (Qiagen, Hilden, Germany). The principle is to use a silica column to adsorb total RNA and genomic DNA in the extract, digest the genomic DNA from the column by the RNAase-free DNAase (Qiagen, Hilden, Germany), and then wash the column with buffers of different salinity to remove DNA, proteins and other contaminants attached in the column. Finally, the total RNA retained on the column was extracted by diethylpyrocarbonate (DEPC)-treated water. The concentration and purity of total RNA were determined by spectrophotometry (NanoDrop, Thermo Fisher Scientific, Wilmington, DE, USA) at wavelengths of 230, 260, 280, and 320 nm.

### 2.7. The Diversity of Microbial Population Composition

Approximately 15 ng of environmental DNA was used as the template for the polymerase chain reaction (PCR) to specifically amplify the hypervariable V3–V4 region of 16S rRNA genes using the high-fidelity DNA polymerase 2× KAPA HiFi HotStart ReadyMix (Roche, Grenzacherstrasse, Switzerland) and the primer set 16SV3V4-F (5′-tcgtcggcagcgtcagatgtgtataagagacAGCCTACGGGNGGCWGCAG-3′) and 16SV3V4-R (5′-gtctcgtgggctcggagatgtgtataagagacAGGACTACHVGGGTATCTAATCC-3′) (the lowercase letters indicate the Illumina adaptor sequences; W=A or T; H=A, T or C; V=A, C or G; N=A, T, G or G). After the attachment of the index sequences, the Illumina MiSeq high-throughput sequencing platform (Illumina, San Diego, CA, USA) was used for amplicon sequence analysis in the pair-end method. The adaptor and primer sequence of high-quality reads (quality score ≤ Q20) were removed by the Cutadapt software (version 5.0) [27]. The diversity of resultant sequences was analyzed by DADA2 analysis [28]. The representative ASV (amplicon sequence variant) obtained by DADA2 analysis was annotated with the 16S rRNA reference sequences in the Silva database (version 138). The indices of richness (abundance-based coverage estimator, ACE) and diversity (Shannon) were calculated by the reads randomly selected from each sample 1000 times and were expressed as averages to avoid biases generated by differences in the sequencing depth.

Regarding the viable microbial population diversity estimation, about one ng of the total RNA was converted into complementary DNA fragments (cDNA) by the SuperScript-III reverse transcriptase (Invitrogen, Waltham, MA, USA) and random oligonucleotides. The cDNA was used to construct a 16S rRNA V3-V4 amplicon library following high-throughput nucleotide sequencing and diversity analysis. All the examination methods were the same as the part of the 16S rRNA genes phylogeny described above.

### 2.8. Analysis of RNA Expression Profiles

The RiboMinus Bacteria Transcriptome Isolation kit (Thermo Fisher Scientific, Wilmington, DE, USA) was applied to remove bacterial rRNA and construct cDNA libraries. After the adaptor and index sequences were attached, the Illumina NovaSeq high-throughput sequencing platform (Illumina, Waltham, MA, USA) was used for sequence analysis in the pair-end method. After the adaptor sequence was removed by the Cutadapt software [27], the clean reads were then de novo assembled to generate the reference transcripts with the Trinity software (version 2.15.0) [29]. The mRNA level of each putative transcript was evaluated by the number of reads precisely mapping the reference transcripts using the RSEM software (version 1.3.3) [30]. Subsequently, the trimmed mean of M values (TMM) algorithm [31] and the edgeR [32] were used to normalize and compare the mRNA level of specific transcripts among all samples. Besides the de novo assembled transcripts, the cDNA sequences of the thiosulfate metabolizing bacteria *Thiomicrorhabdus sediminis* G1 (GCA_005885815) [33] and *Thiomicrorhabdus* marina strain 6S2-11 (NZ_JAGETV010000010) [34] were also selected as another reference for sequence mapping. The physiological functions of putative transcripts annotated against the databases, such as the non-redundant protein database, Gene Ontology (GO), and Kyoto Encyclopedia of Genes and Genomes (KEGG), were carried out by the OmicsBox software (version 3.2) [35].

### 2.9. Sequence Deposition

All the sequences were deposited in the Sequence Read Archive (SRA) database with the accession number PRJNA779824.

## 3. Results

### 3.1. Hydrographic Features

**Figure 2:**
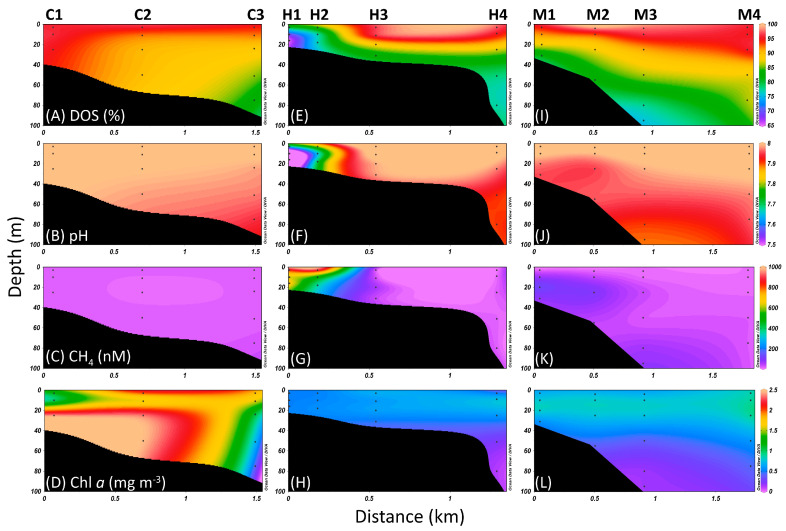
The vertical profiles of (**A**,**E**,**I**) dissolved oxygen saturation (DOS, %), (**B**,**F**,**J**) pH, (**C**,**G**,**K**) the concentrations of methane (CH_4_) and (**D**,**H**,**L**) chlorophyll *a* (Chl *a*) in the waters surrounding the Guishan Islet during the period from 15 to 17 April 2019. The locations of the stations are indicated on the upper side of each column.

Guishan Islet is located in the Kuroshio current (Figure 1). At stations C1 to C2, located in the northwest Guishan Islet, the seawaters had dissolved oxygen saturation (DOS) levels of higher than 90%, pH values of around 8.1, and methane concentrations of below 20 nM (Figure 2A–C, Supplementary Table S1). Diatoms contributed to high concentrations of chlorophyll exceeding 1.5 mg m^−3^ at both stations (Figure 2D). These findings suggested that the Kuroshio water primarily influenced the hydrography in this area. Compared with the northwest region, frequent volcanic-related activities occur on the seafloor of southeast Guishan Islet and have shaped the rare geological scenery, including the shallow-sea hydrothermal vent (Figure 1). The waters in station H1, closest to the hydrothermal vent, had lower pH values, ranging from 7.2 to 7.7; low levels of DOS around 65 to 76%; and high concentrations of CH_4_, ranging from 692 to 827 nM. Additionally, the low chlorophyll *a* concentration of below 0.5 mg m^−3^ indicated a scarcity of larger phytoplankton in station H1. The hydrographic conditions were similar at the H2 station (Figure 2E–H, Supplementary Table S1). In addition to stations H1 and H2, the hydrological characteristics in the waters of the southeast Guishan Islet were influenced by the combination of hydrothermal vent activity and the Kuroshio current. The water retention time here is at most two hours, derived from the high speed of the Kuroshio current [4]. The interaction between the two water masses of the hydrothermal plump and Kuroshio water resulted in the hydrological characteristics found in the stations H3 and H4 and M1 to M4. The deeper waters in these stations were majorly affected by the hydrothermal waters. The mixing effect of the Kuroshio water became more pronounced further away from the hydrothermal field (Figure 2E–L, Supplementary Table S1). Furthermore, at the C3 station, deeper seawater had lower DOS and pH values, likely caused by water intrusion from the hydrothermal plume (Figure 2A–C, Supplementary Table S1).

### 3.2. Distribution of Picoplankton

**Figure 3:**
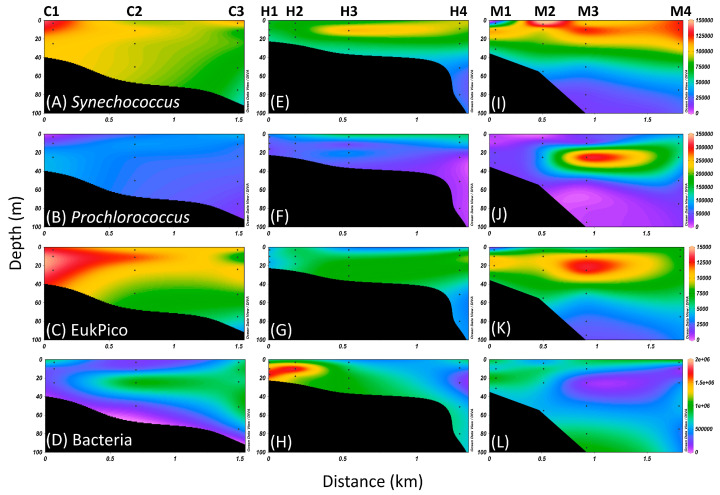
The vertical distributions of (**A**,**E**,**I**) *Synechococcus*, (**B**,**F**,**J**) *Prochlorococcus*, (**C**,**G**,**K**) eukaryotic picophytoplankton, and (**D**,**H**,**L**) heterotrophic bacteria in the waters surrounding the Guishan Islet during the period from 15 to 17 April 2019. The locations of the stations are indicated on the upper side of each column.

Picophytoplankton (cell diameter ≤ 5 μm), including *Synechococcus*, *Prochlorococcus*, and eukaryotic picophytoplankton (Eukpico), represent essential photoautotrophic microorganisms in the subtropical Kuroshio water [36,37]. At stations C1 to C3, where Kuroshio water prevailed, *Synechococcus* and Eukpico exhibited high cell concentrations, exceeding 1 × 10^5^ and 1 × 10^4^ cells mL^−1^, respectively. These concentrations progressively declined toward the open ocean. In contrast, the bacterial abundance followed an opposite pattern, starting from fewer than 5 × 10^5^ cells mL^−1^ at station C1 and slightly increasing to about 1 × 10^6^ cells mL^−1^ at station C3. The *Prochlorococcus* population in this area was comparatively lower, with an abundance of less than 0.5 × 10^5^ cells mL^−1^ (Figure 3A–D). At the stations closest to the hydrothermal vent (H1 and H2), there was a noticeable decrease in *Synechococcus*, *Prochlorococcus*, and Eukpico abundance. As the distance from the vent increased, the abundance of these picophytoplankton progressively recovered (Figure 3E–G). This observation suggested that hydrothermal plume had an unfavorable impact on the growth and survival of picophytoplankton populations. Unlike the distribution of photosynthetic organisms, bacterial abundance peaked at hydrothermal vent stations H1 and H2, exceeding 1.5 × 10^6^ cells mL^−1^ (Figure 3H). Due to the combined influence of hydrothermal plump and Kuroshio waters, the picophytoplankton thrived in the upper layers at stations M1 to M4. In particular, *Prochlorococcus* reached its highest abundance of more than 3.5 × 10^5^ cells mL^−1^ in the subsurface of station M3 (Figure 3I–K). In the deeper waters at stations M1 and M2, the bacterial abundances were approximately 1 × 10^5^ cells mL^−1^, comparable to those at stations C2 and C3, but remained lower than the bacterial levels observed at stations H1 and H2 (Figure 3L).

### 3.3. Dissolved Inorganic Carbon (DIC) Fixation Efficiency

**Figure 4:**
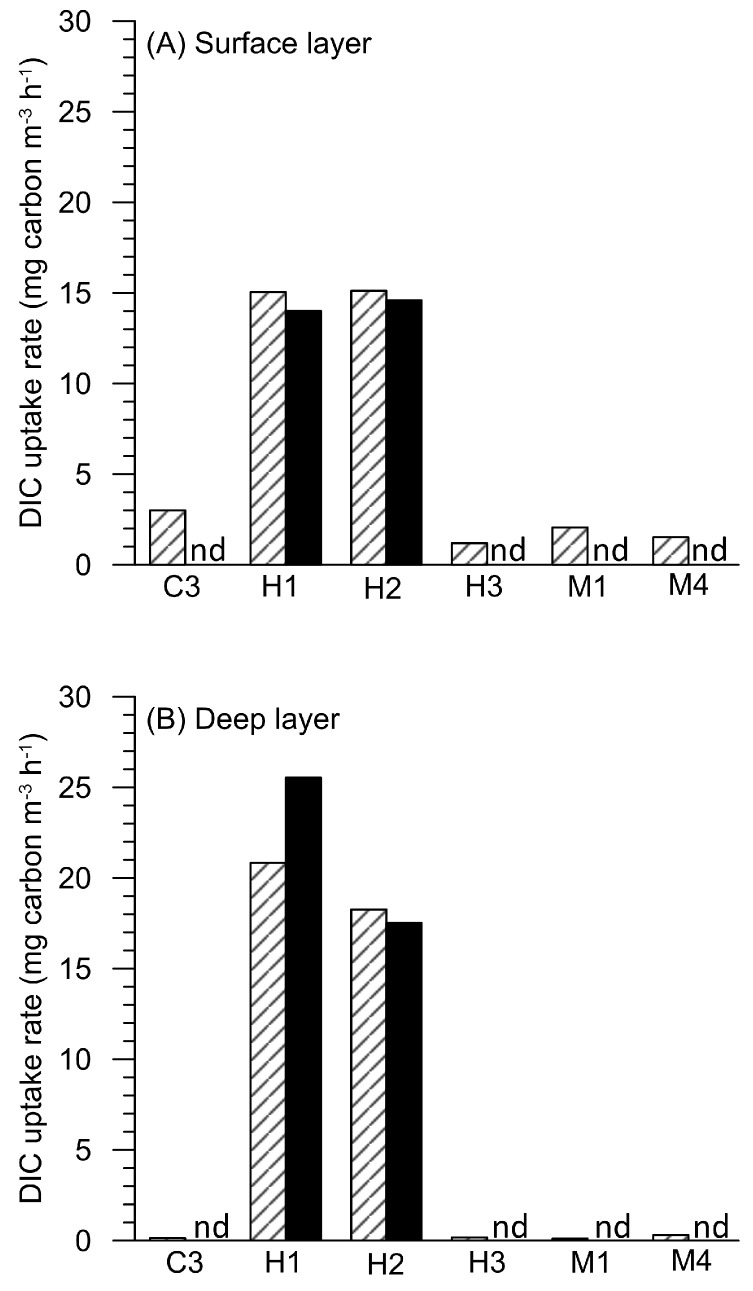
The dissolved inorganic carbon (DIC) fixation efficiency in the waters of (**A**) surface and (**B**) deep layers surrounding the Guishan Islet during the period from 15 to 17 April 2019. The data retrieved from the samples incubated under the light intensity at 2200 μmole photons m^−2^ s^−1^ and in the dark are indicated as white and black bars. “nd” means “non-detectable”.

The DIC uptake rates in different waters surrounding Guishan Islet are shown in Figure 4. Under a light intensity of 2000 μmole photons m^−2^ s^−1^, the DIC uptake rates in the surface and deep waters of the C3 site are 3 and 0.14 mg carbon m^−3^ h^−1^, respectively. Similar results were obtained from measurements at stations H3, M1, and M2. No DIC uptake activity under dark conditions was detected at these stations. However, at stations H1 and H2, vigorous DIC uptake activity was observed in the water samples in the dark. At the H1 site, the DIC uptake rates of surface and deep waters in the dark were 14.01 mg carbon m^−3^ h^−1^ and 25.53 mg carbon m^−3^ h^−1^, respectively. Similar high efficiencies of dark DIC uptake were observed at the H2 station, with measurements of 14.59 mg of carbon m^−3^ h^−1^ at the surface and 17.52 mg of carbon m^−3^ h^−1^ in the deep layer. At both these stations, the DIC uptake efficiency under the light condition was equal to the dark treatment. This suggests that the primary productivity in the waters influenced by hydrothermal vent plumes was mainly driven by chemolithoautotrophs (Figure 4).

### 3.4. Diversity of Prokaryotic Picoplankton Community Composition

**Figure 5:**
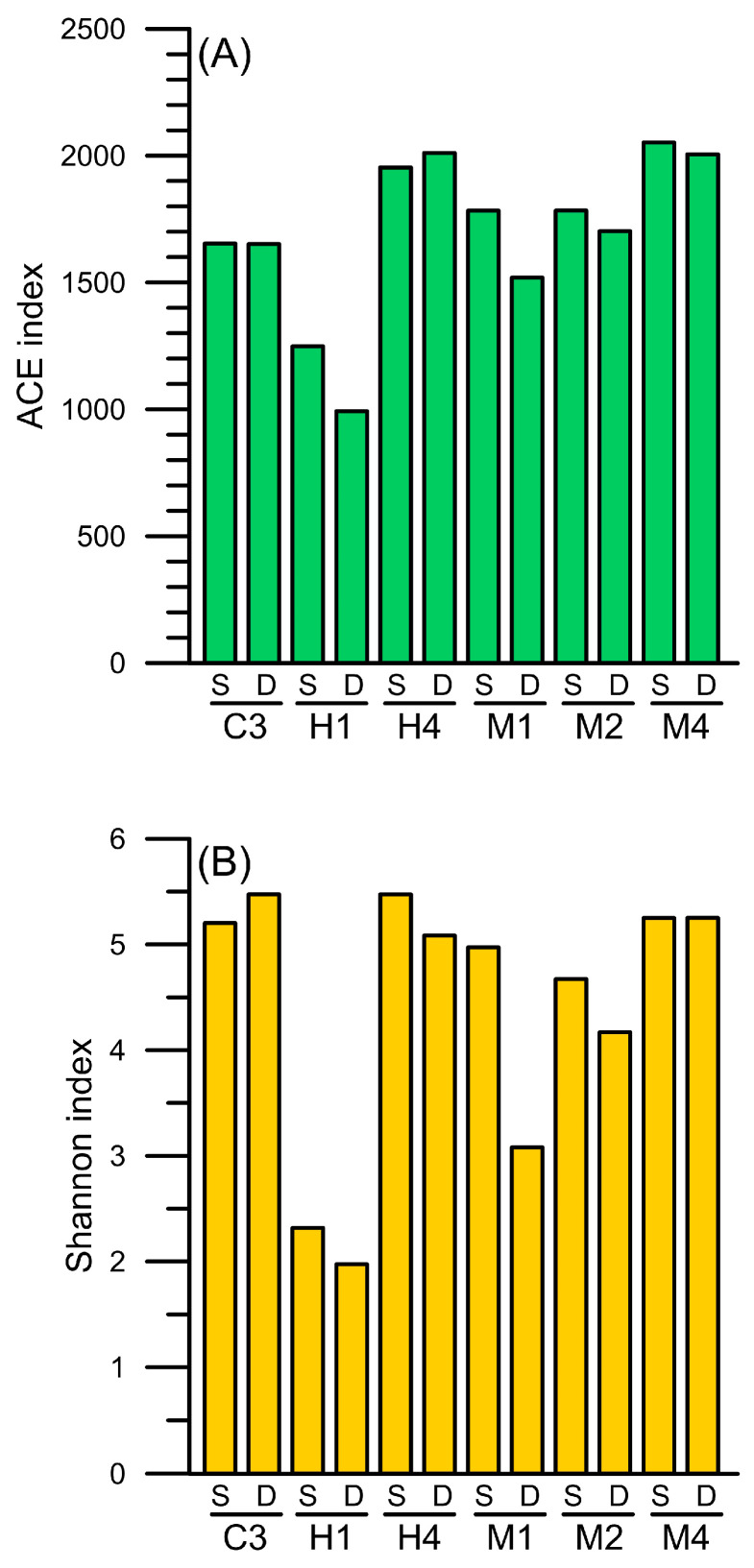
The (**A**) abundance-based coverage estimator (ACE) and (**B**) Shannon diversity indices of the community assemblages of prokaryotic picoplankton, which were inferred from the ASV composition based on the V3 to V4 region of 16S rRNA gene (16S rDNA) sequences in the waters surrounding the Guishan Islet during the period from 15 to 17 April 2019. The letters S and D indicate the surface and deep layer samples, respectively.

Using the V3–V4 fragments of 16S rRNA gene (16S rDNA) as a phylogeny indicator of prokaryotic picoplankton assemblage composition, 5186 ASVs were identified in all samples (see Supplementary Table S2). At station C3, the farthest from the hydrothermal vent area, the microbial composition exhibited a richness of approximately 1600, as measured by the ACE index, and a diversity of about 5, as indicated by the Shannon index. At station H1, the microbial compositions in the surface and deep layers showed the lowest ACE index at approximately 1000, along with a Shannon diversity index of 2. In addition to H1, a low Shannon index value was observed in the deep water at station M1. The low diversity index observed in the waters affected by the hydrothermal plume implied the presence of dominant microbial species (Figure 5).

**Figure 6:**
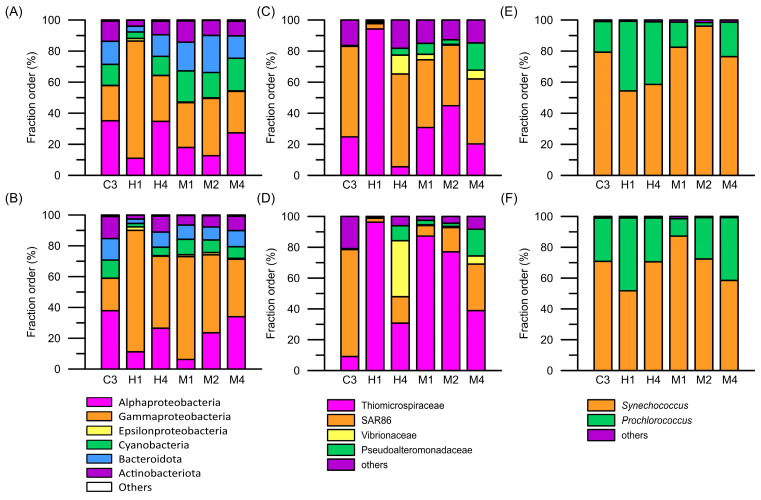
The assemblage composition of prokaryotic picoplankton, (**A**,**B**) total prokaryotic picophytoplankton (in phylum level), (**C**,**D**) *Gammaprobacteria*, and (**E**,**F**) picocyanobacteria, in the waters surrounding the Guishan Islet during the period from 15 to 17 April 2019, which were inferred from the V3 to V4 region of 16S rRNA gene (16S rDNA) sequences. The upper panels (**A**,**C**,**E**) and lower panels (**B**,**D**,**F**) show the compositions of prokaryotic picoplankton in the surface and deep layers, respectively.

The microbial composition in the surface and deep layers at station C3 was evenly made up of *Alphaproteobacteria*, *Gammaproteobacteria*, *Cyanobacteria*, *Bacteroidota*, and *Actinobacteriota* (Figure 6A,B). In contrast, at station H1, closest to the hydrothermal vents, the proportion of *Gammaproteobacteria* was as high as 80% in both the surface and bottom layers. Furthermore, *Gammaproteobacteria* were found in the deeper layers at the other stations affected by the hydrothermal vent plume. *Gammaproteobacteria* comprised over 60% of the microbial community in the deep layer at station M1. In the deep layers of other stations H4, M2, and M4, which were far from the hydrothermal vent, the proportion of *Gammaproteobacteria* exceeded 30% (Figure 6A,B). In analyzing the composition of *Gammaproteobacteria* in the waters influenced by the hydrothermal vent plume, the primary group identified was *Thiomicrospiraceae*, which was exclusively composed of the genus *Thiomicrorhabdus* (Figure 6C,D). However, these composition patterns differed from those observed at station C3, where *Gammaproteobacteria* was predominately composed of the SAR86 cluster (Figure 6C,D). Another dominant community was *Cyanobacteria*, comprising *Synechococcus* WH8102 (clade-III) and *Prochlorococcus* PCC9511 (High Light-II clade). Aside from their varied distributions, there were no differences in population composition across all stations (Figure 6E,F). Moreover, previous studies suggested that *Epsilonproteobacteria* were the dominant bacteria in hydrothermal vents. In the study area, *Sulfurimonadaceae* and *Sulfurovaceae* were the major members of *Epsilonproteobacteria*. However, our findings indicate that *Epsilonproteobacteria* comprised less than 5% of the bacterial community composition at station H1 and were not detected at any other stations (Figure 6A,B).

**Figure 7:**
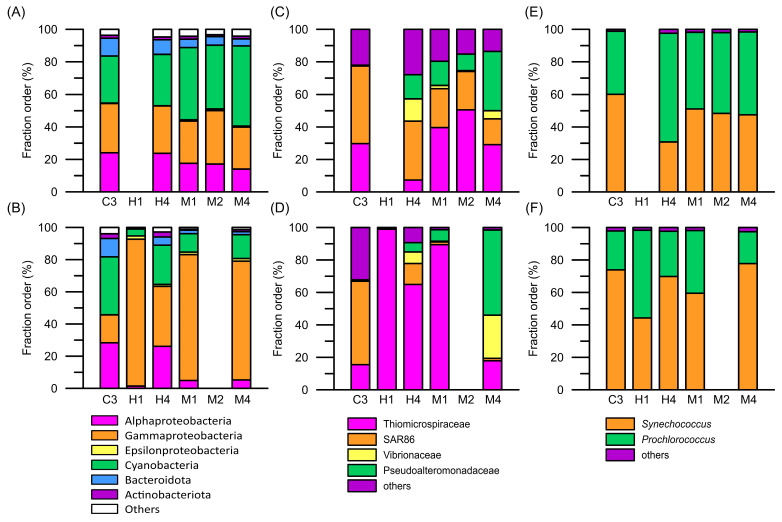
The assemblage composition of active prokaryotic picoplankton, (**A**,**B**) total active prokaryotic picophytoplankton (in phylum level), (**C**,**D**) *Gammaprobacteria*, and (**E**,**F**) picocyanobacteria, in the waters surrounding the Guishan Islet during the period from 15 to 17 April 2019, which were inferred from the V3 to V4 region of 16S rRNA (16S rRNA) sequences. The upper panels (**A**,**C**,**E**) and lower panels (**B**,**D**,**F**) show the compositions of prokaryotic picoplankton in the surface and deep layers, respectively.

The V3–V4 fragments of 16S rRNA (16S rRNA) were also used to indicate cell viability to evaluate the active microbial community structure. The active prokaryotic picoplankton community structures resembled those obtained from the previous 16S rDNA analysis (Figure 7). In the deep waters of stations H1 and M1, *Thiomicrospiraceae* was still a vigorous microbial community. However, the results from the study show a distinct difference between the 16S rDNA and 16S rRNA analyses. While the proportion of Cyanobacteria in the 16S rDNA analysis was relatively low, at about 10% at most, the 16S rRNA analysis revealed a different result. Except for station H1, the proportion of active cyanobacteria in the surface water at the other stations exceeded 20%. At station M4, this proportion reached as high as 40%, with *Prochlorococcus* PCC9511 (High Light-II clade) being the predominant active picocyanobacterium (Figure 7).

**Figure 8:**
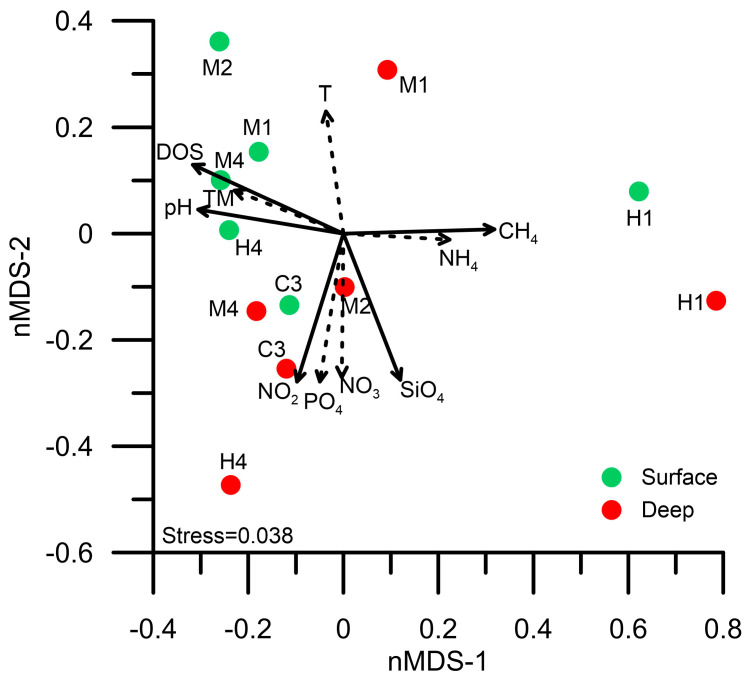
The nonmetric multidimensional scaling (nMDS) ordination analysis of the community composition of prokaryotic picoplankton in the waters surrounding the Guishan Islet from 15 to 17 April 2019. The correlation relationships between the picoplankton assemblages and environmental factors among all stations were assessed with the function “envfit” (number of permutations = 999) in the R package “vegan”. The solid and dashed lines indicate significance (*p*-value) at the ≤0.01 and ≤0.05 levels, respectively. T, temperature; TM, turbidity; DOS, dissolved oxygen saturation.

Figure 8 illustrates the beta diversity analysis of 16S rDNA samples from each station and their correlations with ambient hydrological factors. The microbial compositions in both the surface and deep layers at station H1 were similar. They exhibited positive relationships with concentrations of methane (CH_4_) (*p* ≤ 0.01) and ammonium (NH_4_) (*p* ≤ 0.05) and negative relationships with pH (*p* ≤ 0.01), dissolved oxygen saturation (DOS) (*p* ≤ 0.01), and light transmission (TM) (*p* ≤ 0.01). These results suggest that the hydrothermal vent plume mainly influenced the microbial compositions in station H1 (Figure 8). Excluding station H1, the differences in beta diversity among the other stations can be categorized into two groups based on water depth. In the surface waters of stations H4, M1, M2, and M4, the microbial community compositions displayed positive relationships with pH (*p* ≤ 0.01), DOS (*p* ≤ 0.01), and TM (*p* ≤ 0.01). By contrast, the beta diversities in both surface and deep layers at station C3, as well as in the deep waters at stations H4 and M4, showed positive correlations with the concentrations of various inorganic nutrients (i.e., NO_2_, NO_3_, PO_4_, and SiO_4_) (Figure 8).

### 3.5. Gene Expression Profiles of Prokaryotic Picoplankton

**Figure 9:**
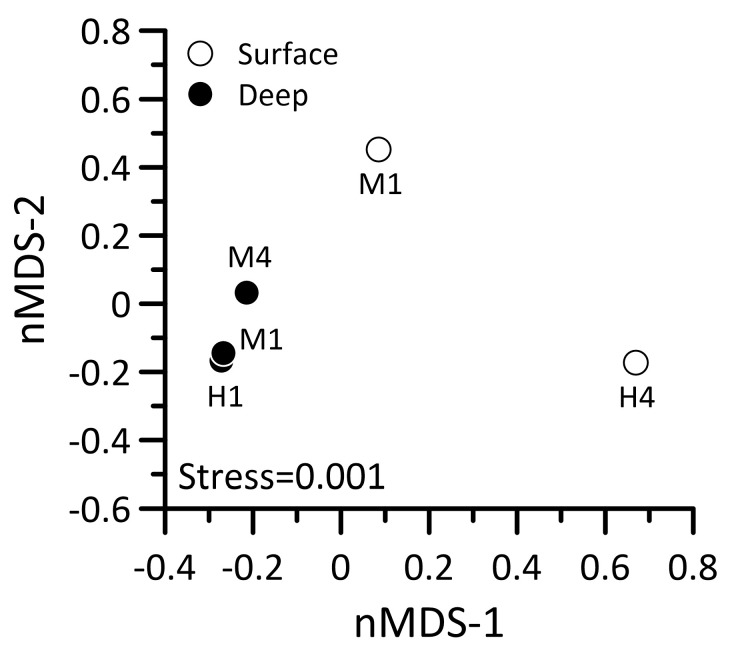
The nMDS ordination analysis of the transcript compositions of prokaryotic picoplankton in the waters surrounding the Guishan Islet during the period from 15 to 17 April 2019.

**Figure 10:**
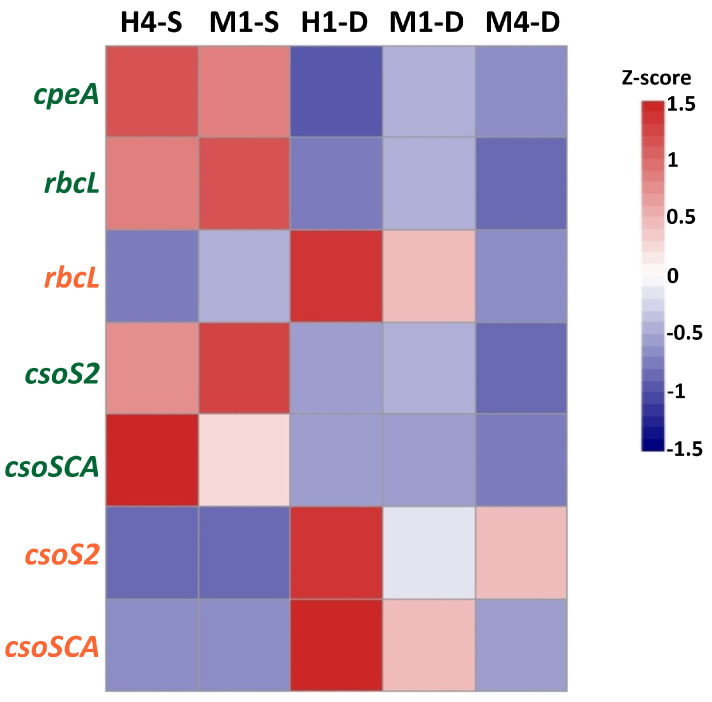
The comparison of carbon fixation-related gene expression across the surface and deep waters of different stations near the hydrothermal vent of Guishan Islet from 15 to 17 April 2019. Gene names in green or orange denote their affiliation with either picocyanobacteria or *Thiomicrorhabdus*. The difference in the expression of each gene is expressed as a z-score. Red represents upregulation, and blue represents downregulation. The letters S and D indicate the samples obtained from the surface and deep layer, respectively.

To understand how the hydrothermal vent plume interacts with the surrounding seawater and its effects on microbial physiology, we performed a metatranscriptome analysis to study the dynamics of microbial gene expression in the waters near the hydrothermal vent field. Two samples were collected from the surface layer at stations M1 and H4, and three were collected from the deep layer at stations H1, M1, and M4 (Figure 1). The nMDS (non-metric multidimensional scaling) analysis revealed that the microbial transcript compositions in the deep waters at stations H1 and M1 were highly similar. Additionally, the microbial transcript composition in the deep water at station M4 showed comparable characteristics to those observed at stations H1 and M1. In contrast, the transcript compositions in the surface waters of stations M1 and H4 exhibited significant differences from those in the deep waters (Figure 9). Upon examining the transcripts expressed at these two stations, it was evident that most of them are associated with photosynthesis by picocyanobacteria. For instance, the gene for the alpha subunit of phycoerythrin (*cpeA*) was significantly upregulated. Additionally, the genes related to the carboxysome-mediated carbon dioxide-concentration mechanism (carboxysome-CCM) and the Calvin–Benson–Bassham cycle (CBB), including *csoS2* (carboxysome-related protein), *csoSCA* (carboxysome carbonic anhydrase), and *rbcL* (large subunit of ribulose-1,5-bisphosphate carboxylase/oxygenase), showed similar expression patterns in the surface waters of both stations (Figure 10).

**Figure 11:**
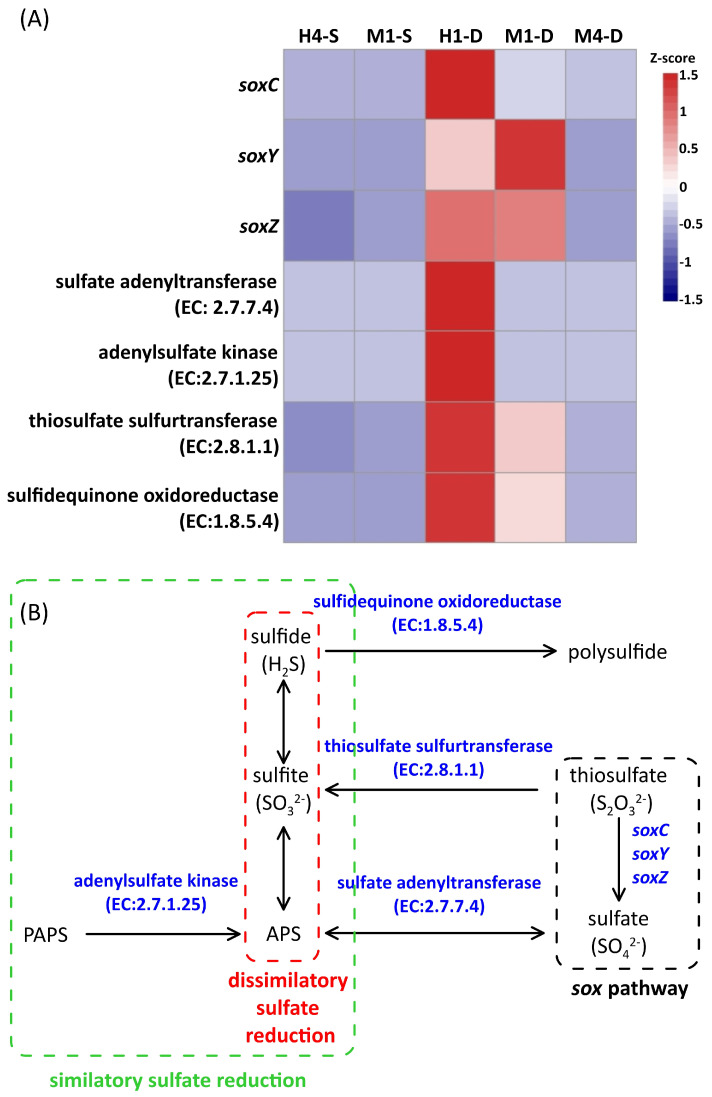
(**A**) The comparison of sulfur metabolism-related gene expression in *Thiomicrorhabdus* cells across the surface and deep waters of different stations located near the hydrothermal vent of Guishan Islet from 15 to 17 April 2019. The difference in expression of each gene is expressed as a z-score. Red represents upregulation, and blue represents downregulation. The letters S and D indicate the samples obtained from the surface and deep layer, respectively. (**B**) Schematic representation of the locations of the genes shown in panel (**A**) within the sulfur metabolism pathway [34]. APS: adenosine phosphosulfate; PAPS: phosphoadenosine phosphosulfate.

The proportion of reads attributed to *Thiomicrorhabdus* in each sample varied significantly, ranging from a high of 29.8% in the sample from the deep water of station H1 to a low of 0.5% in the surface samples of stations H4 and M1 (see Supplementary Table S3). To confirm that *Thiomicrorhabdus* is the chemolithotroph contributing to considerable primary production in the hydrothermal vent area of Guishan Islet, its transcripts related to the carbon dioxide fixation processes were examined. Our analysis showed that the carboxysome-related genes, including *csoS2* and *csoSCA*, and the CBB-related gene *rbcL*, were up-regulated in the deep waters of stations H1 and M1. The highest expression levels of these genes were recorded at station H1, which is closest to the hydrothermal vent (Figure 10). The energy source for carbon fixation in *Thiomicrorhabdus* derives from the oxidation of reduced sulfur compounds. The SOX (sulfur-oxidizing) system is a critical sulfur oxidation pathway for converting thiosulfate to sulfate. The genes *soxC*, *soxY*, and *soxZ*, which encode enzymes involved in thiosulfate oxidation, were significantly expressed at the deep waters of stations H1 and M1 (Figure 11). In addition to the SOX pathway, the genes encoding sulfate adenylyltransferase (EC:2.7.7.4), adenylylsulfate kinase (EC:2.7.1.25), and thiosulfate sulfurtransferase (EC:2.8.1.1) show high expression levels in the deep water at stations H1 and M1 (Figure 11). The gene encoding sulfidequinone oxidoreductase (EC:1.8.5.4), which facilitates the conversion of hydrogen sulfide (H_2_S) into inorganic polysulfide (H_2_Sn), was also found to be highly expressed in the deep waters at stations H1 and M1 (Figure 11). It was found that the dissimilatory sulfate reduction pathway, which converts thiosulfate to adenosine 5′-phosphosulfate (APS) or to polysulfide ([H_2_S]n), occurred in *Thiomicrorhabdus* cells.

## 4. Discussion

The waters affected by the hydrothermal vent plume had low chlorophyll and picophytoplankton concentrations but had high bacterial abundance (Figure 2 and Figure 3). High DIC uptake rates were also observed in these waters under the dark treatment (Figure 4). This indicated that chemolithotrophs mainly contributed to primary productivity in the hydrothermal vent field off Guishan Islet in the late spring. The contribution of chemolithotrophs to primary production was about 15 to 25 mg carbon m^−^^3^ h^−^^1^ in the hydrothermal vent field of Guishan Islet, which was consistent with Lin et al. (2021) [4] but much higher than the 0.1 to 1 mg carbon m^−^^3^ h^−^^1^ in other shallow-sea hydrothermal areas [5,6]. These variations in dark DIC fixation may result from spatial heterogeneity in carbon fixation activity (Figure 2) [4]. In addition to the carbon fixation by chemolithotrophs, picophytoplankton contributed less than 10% of primary production in the waters above the hydrothermal vents. The distribution of sulfide-tolerant photosynthesis within the hydrothermal plume requires further exploration. In this hydrothermal field, photosynthesis and chemolithotrophy occurred simultaneously; however, chemolithotrophs were the dominant group, driving nearly all primary productivity. A significant portion of the organic material produced was transported downward, supporting a diverse range of organisms adapted to the toxic and extreme conditions of the vents. This highlights the crucial role of chemolithotrophy in sustaining life in such extreme ecosystems [38].

It is recognized that the influx of nutrients from deep water due to upwelling is essential for phytoplankton growth in the Kuroshio current [39,40]. Besides the upwellings, the nutrients from the hydrothermal plume should also be acknowledged. Compared to the high dark DIC fixation rates at stations H1 and H2, the primary productivity in the surface waters of the outside-vent stations was contributed by larger photoautotrophs (e.g., *Chaetoceros* spp. found at station C3), ranging from 1 to 3 mg carbon m^−^^3^ h^−^^1^. These values were slightly higher than the primary productivity of approximately 0.6 mg carbon m^−^^3^ h^−^^1^ in the surface water of subtropical Kuroshio during the late spring [39]. This suggests that the nutrients from the hydrothermal vent also support increased carbon fixation from photosynthesis around Guishan Islet. Furthermore, Lin et al. (2020) indicated that frequent tidal eddies resulted in water residence time of about 1 to 2 h in the hydrothermal area in Guishan Islet [41]. Such results suggest that the nutrient-rich water derived from the hydrothermal vent injection are rapidly transported northeastward along with the Kuroshio current. The extent of the contribution of these nutrients to the growth of phytoplankton and the biogeochemical cycle in the Kuroshio current still needs to be clarified in future research.

CO_2_-concentrating mechanisms (CCMs) enhance the concentration of CO_2_ in the vicinity of ribulose bisphosphate carboxylase/oxygenase (Rubisco), thereby reducing the competitive inhibition by O_2_ and improving the efficiency of CO_2_ fixation. Carboxysomes, a component of CCMs present in all cyanobacteria and certain proteobacteria, encapsulate CO_2_-fixing enzymes such as Rubisco and carbonic anhydrase within a polyhedral protein shell [42,43,44,45]. Scott et al. (2019) pointed out that carboxysomes were lacking in *Thiomicrorhabdus* sp. strain Milos-T2 and *Thiomicrorhabdus arctica* based on electron microscopy observations and genome data [46]. On the contrary, the genome data showed that *Thiomicrorhabdus sediminis* possessed carboxysome-related genes [33]. Such genetic differences suggested the high diversity of the genus *Thiomicrorhabdus*. Our metatranscriptomic analysis indicated the expression of genes encoding carboxysome-involving carbon fixation enzymes in *Synechococcus* and *Thiomicrorhabdus* exhibited distinct regional distribution patterns (Figure 10). Carboxysomes help these microorganisms to efficiently capture and concentrate CO_2_ in marine environments, even when CO_2_ levels are low [47]. Different autotrophs inhabiting various ecological niches jointly contributed to the primary production at the shallow-sea hydrothermal vent by employing similar CCM. This synergistic effect enhanced carbon sequestration and supports the functioning of the food chain in this region.

Although the concentration of H_2_S was not measured in this study, previous reports indicated that the concentration was below 0.8 mg L^−^^1^ in the hydrothermal plume at Guishan Islet [11]. Zhang et al. (2012) [11] and Li et al. (2018) [3] indicated that two dominant sulfur metabolism-related chemolithotrophs were present in different locations near hydrothermal vents. *Nautilia*, affiliated with *Epsilonproteobacteria*, was found around the vents, while *Thiomicrospira*, affiliated with *Gammaproteobacteria*, occupied the water bodies farther away from the vents [3,11]. Our findings were almost consistent with their reports. However, we did not collect the vent fluid because we focused on the microbial community composition influenced by the hydrothermal plumes. Therefore, *Epsilonproteobacteria* constituted only a tiny proportion of these samples. It was possibly due to rapid mixing with hydrothermal vent plumes and Kuroshio water. Instead, another variant of dominant thiosulfate-oxidizing bacteria, *Thiomicrorhabdus*, was found. The genus *Thiomicrorhabdus* currently comprises eleven species, four of which were reclassified from the genus *Thiomicrospira* in 2017. All members of *Thiomicrorhabdus* are obligate chemolithoautotrophic sulfur-oxidizing bacteria. They can utilize inorganic sulfur compounds, such as thiosulfates and sulfides, for their growth [48]. The SOX pathway is one of the molecular mechanisms for oxidizing thiosulfate into sulfate [47]. Based on the genomic annotation, *Thiomicrorhabdus* possesses essential genes that participate in the SOX pathway operation. The genome analysis and physiological experiments have confirmed that the SOX system is the only molecular mechanism for thiosulfate oxidation in *Thiomicrorhabdus* [34]. Unlike the SOX system, which operated under aerobic conditions, the genes participating in the dissimilatory sulfate reduction occurring under hypoxic conditions were also found in *Thiomicrorhabdus* [34]. This suggests that thiosulfate can be reduced to sulfite, which can then either be transformed into the organic sulfur compound APS or converted into polysulfides (H_2_Sn) by sulfide/quinone oxidoreductase (EC 1.8.5.4) under anoxygenic conditions. However, unlike the SOX pathway, the dissimilatory sulfate reduction usually occurs under anoxygenic conditions [34]. Our study first presented the transcript distribution of thiosulfate metabolism-related genes in *Thiomicrorhabdus* in the waters near a shallow-hydrothermal field. These genes showed significant expression in waters with acidic conditions, low dissolved oxygen saturation, and high CH_4_ concentrations (Figure 2 and Figure 8). Consequently, in the hydrothermal field, the operation of the SOX pathway and the dissimilatory sulfate reduction in *Thiomicrorhabdus* served a dual purpose, contributing to energy production and detoxification. Additionally, they played a crucial role in driving the biogeochemical sulfur cycle through microbial activity.

*Epsilonproteobacteria* are known to dominate bacterial communities in deep-sea hydrothermal vents, comprising 66–98% of the total bacterial population. They play a crucial role in these environments’ nitrogen and sulfur cycles [49]. In the shallow-sea hydrothermal vent at Guishan Islet, *Epsilonproteobacteria* remained the dominant species, accounting for up to 80% of the total bacterial community within a 3 m radius of the vents. However, beyond this range, the dominant bacterial group shifted to *Gammaproteobacteria*, which comprised up to 90% of the community [11]. Our findings, however, indicate that *Epsilonproteobacteria* constituted less than 5% of the bacterial community composition at station H1 and were undetectable at other stations. This study focused on the effects of hydrothermal plumes on microbial composition. The distribution of bacterial communities suggested that *Epsilonproteobacteria* are better adapted to the extreme conditions near hydrothermal vents. Nevertheless, when environmental pressure was alleviated, *Gammaproteobacteria* became the dominant group. Measuring primary productivity typically requires short-term culturing, but simulating the extreme conditions of hydrothermal vents remains challenging. As a result, assessing the primary productivity of *Epsilonproteobacteria* in situ is still tricky. Advances in in situ measurement technologies, such as nanoSIMS (nanoscale secondary ion mass spectrometry), may provide a feasible solution to this challenge.

## 5. Conclusions

Many shallow-water hydrothermal systems are located on the exposed coasts of tiny volcanic islands, making them well-flushed by surface currents, with water residence times as short as a few hours [4,40]. Thus, the hydrography in Guishan Islet is highly dynamic, resulting in the joint influence of the hydrothermal vent plume and the Kuroshio current. The relationship between microbial ecology and biogeochemical cycles in this area is still an open question. While several previous studies have presented valuable information on this issue [3,4,11], the simultaneous exploration of the hydrology, the primary productivity, the microbial community composition, and their genetic expression remains lacking in this area. In this study, combining the hydrography survey, the DIC fixation efficiency measurement, and the omics analysis, we revealed the thiosulfate oxidizing bacteria, *Thiomicrorhabdus*, exclusively contributed to the primary production in the waters mixing with the hydrothermal vent plume. In addition to chemolithotrophs, picocyanobacteria were the primary producers in the waters out of the hydrothermal vent. Both of them sustained the operation of the shallow-sea hydrothermal ecosystem. Even though we completely understand the microbial ecology and primary productivity in the hydrothermal vents around Guishan Islet, the functioning of the biogeochemical cycle still needs to be further understood, especially the carbon cycle. Although we had also analyzed the 18S rRNA gene phylogeny of nano-protists (cell size ≤ 5 μm), there were no significant differences in their community composition among all samples. It was proposed that primary consumers might possess the ability to withstand the toxic substances present in the hydrothermal fluid. The carbon cycling process via the biological carbon pump mechanism still needs to be explored. Furthermore, the nutrients and carbon flux transferred laterally from the hydrothermal vent area of Guishan Islet to the Kuroshio current, due to the short water residence time, require accurate evaluation. Our results highlight the significance of further studies on how hydrothermal vents affect marine ecosystems and biogeochemical cycles to improve our comprehension of oceanic environmental processes. 

## Data Availability

All the sequences were deposited in the Sequence Read Archive (SRA) Database with the accession number PRJNA779824.

## Supplementary Materials

The following supporting information can be downloaded at https://www.mdpi.com/article/10.3390/biology14010028/s1. Table S1: The hydrography of Guishan Islet during the period from 15 to 17 April 2019; Table S2: The sequencing data of the 16S rRNA gene V3-V4 amplicons (16S rDNA) obtained from Guishan Islet during the period from 15 to 17 April 2019; Table S3: The sequencing data of the RNAseq obtained from the waters near the hydrothermal vent of Guishan Islet during the period from 15 to 17 April 2019.
