# Supplementary material for: Dominance of Sulfur-Oxidizing Bacteria, Thiomicrorhabdus, in the Waters Affected by a Shallow-Sea Hydrothermal Plume
Source: Biology (Basel). 2025 Jan 1;14(1):28. doi: 10.3390/biology14010028 (PMC11763282; doi:10.3390/biology14010028)
Supplement: Supplementary file 1 [file biology-14-00028-s001.zip › SuppTableS1_Ksd_Hydrography_Apr2019.pdf]

Supplementary Table S1. The hydrography of Guishan Islet during the period from 15 to 17 April 2019. (TM: particle transmission; DOS: dissolved oxygen saturation; Chl. *a*: chlorophyll *a*)

Station	Longitude (°E)	Latitude (°N)	Bottom depth (m)	Sampling depth (m)	Temperature (°C)	Salinity	TM (%)	pH	DOS (%)	NH_4 (μM)	NO_2 (μM)	NO_3 (μM)	PO_4 (μM)	SiO_4 (μM)	Chl *a* (mg m^{-3})	CH_4 (nM)
C1	121.9414	24.8535	35	3	23.473	34.595	92.8	8.020	95.9	0.3	0.49	0.4	0.10	2.6	1.184	23.49
				10	23.391	34.597	93.1	8.024	96.2	0.3	8.53	1.4	0.13	2.6	0.975	24.73
				25	23.279	34.597	93.6	8.019	95.0	0.3	6.72	1.1	0.14	2.6	2.399	24.41
C2	121.9439	24.8587	65	3	23.525	34.597	93.2	8.023	95.8	0.3	5.60	0.7	0.13	3.3	2.026	21.02
				11	22.867	34.608	93.7	8.009	92.3	0.2	3.71	2.1	0.20	3.9	1.586	18.40
				25	22.329	34.597	94.5	8.005	90.6	0.5	10.68	2.8	0.25	5.0	2.381	17.47
				50	21.607	34.603	95.0	7.985	90.3	0.3	1.62	2.0	0.27	5.6	2.487	20.93
C3	121.9453	24.8657	197	3	23.389	34.599	93.8	8.026	94.4	0.2	0.62	0.6	0.08	1.9	1.804	25.16
				11	23.012	34.608	94.3	8.013	92.3	0.3	0.48	0.9	0.12	2.9	1.594	23.04
				24	22.246	34.604	94.7	7.987	89.5	0.4	0.50	5.3	0.24	2.9	1.063	22.75
				51	21.134	34.609	94.7	7.974	85.9	0.2	1.01	3.4	0.34	7.0	0.719	22.58
				75	20.119	34.604	94.7	7.948	82.5	0.1	0.58	4.1	0.43	8.8	0.400	32.45
				101	18.656	34.613	94.6	7.913	76.0	0.3	1.37	8.3	0.55	17.8	0.236	28.16

Supplementary Table (Continued)

Station	Longitude (°E)	Latitude (°N)	Bottom depth (m)	Sampling depth (m)	Temperature (°C)	Salinity	TM (%)	pH	DOS (%)	NH_4 (μM)	NO_2 (μM)	NO_3 (μM)	PO_4 (μM)	SiO_4 (μM)	Chl *a* (mg m^{-3})	CH_4 (nM)
H1	121.9669	24.8353	19	3	24.666	34.517	95.0	7.669	80.9	0.5	2.19	1.0	0.08	3.8	0.519	827.12
				10	23.819	34.551	88.7	7.311	69.8	0.5	6.51	1.7	0.15	5.7	0.521	636.41
				16	22.748	34.583	90.1	7.174	65.3	0.5	0.37	1.2	0.11	6.4	0.519	691.75
H2	121.9685	24.8359	22	3	24.501	34.522	95.6	7.799	84.2	0.2	7.48	1.6	0.13	3.3	0.521	919.59
				10	23.810	34.544	92.4	7.646	76.9	0.3	3.35	0.8	0.10	4.4	0.537	512.98
				18	22.878	34.582	93.4	7.724	77.1	0.3	10.58	2.0	0.11	4.8	0.564	357.91
H3	121.9720	24.8367	38	3	25.147	34.496	96.5	8.053	96.8	0.3	4.22	0.2	0.05	1.3	0.672	10.48
				11	25.142	34.509	96.6	8.027	97.0	0.3	3.59	0.8	0.08	2.0	0.600	34.07
				20	23.411	34.586	96.4	7.986	88.7	0.3	8.80	2.0	0.13	3.3	0.631	71.01
				31	20.501	34.643	94.9	7.985	82.3	0.3	0.77	3.5	0.28	5.9	0.482	38.43
H4	121.9794	24.8364	134	3	25.445	34.497	96.6	8.052	97.3	0.2	3.48	0.0	0.04	1.2	0.505	6.79
				9	24.555	34.545	96.6	8.024	95.4	0.3	5.81	1.4	0.11	2.9	0.744	16.41
				25	22.149	34.611	95.1	7.974	87.1	0.2	11.98	4.1	0.33	6.6	0.593	23.71
				51	18.885	34.613	93.7	7.916	78.3	0.3	0.99	5.8	0.58	10.4	0.241	20.12
				80	18.610	34.616	93.7	7.910	77.3	0.2	5.26	6.4	0.58	10.6	0.121	24.62
				119	18.197	34.623	93.8	7.901	76.5	0.3	5.88	6.8	0.61	10.5	0.150	22.47

Supplementary Table (Continued)

Station	Longitude (°E)	Latitude (°N)	Bottom depth (m)	Sampling depth (m)	Temperature (°C)	Salinity	TM (%)	pH	DOS (%)	NH_4 (μM)	NO_2 (μM)	NO_3 (μM)	PO_4 (μM)	SiO_4 (μM)	Chl *a* (mg m^{-3})	CH_4 (nM)
M1	121.9568	24.8330	34	3	24.544	34.541	95.1	8.034	94.8	0.4	1.67	0.4	0.09	1.8	0.689	21.75
				10	24.313	34.555	95.2	8.008	91.7	0.3	0.52	0.4	0.10	2.3	0.777	75.05
				20	23.936	34.564	94.5	7.973	88.2	0.3	1.80	1.2	0.09	3.1	0.824	127.58
				31	20.580	34.635	95.0	7.957	81.2	0.3	2.45	2.3	0.20	5.3	0.577	84.81
M2	121.9582	24.8291	60	4	24.631	34.540	95.5	8.036	98.8	0.3	1.31	0.3	0.10	2.1	0.726	21.11
				10	23.986	34.567	95.4	7.994	93.2	0.4	0.61	0.8	0.14	2.6	0.808	44.91
				25	23.331	34.575	95.0	7.952	87.9	0.4	0.51	1.2	0.16	3.9	0.769	104.45
				55	19.884	34.629	94.9	7.965	81.9	0.4	1.02	3.3	0.41	7.3	0.397	44.26
M3	121.9564	24.8258	105	4	24.033	34.561	95.4	8.038	97.5	0.4	0.77	0.6	0.11	2.2	0.691	15.14
				10	23.867	34.565	95.4	8.034	96.6	0.3	0.18	0.5	0.09	2.1	0.785	14.79
				25	22.644	34.589	95.2	7.991	91.1	0.3	5.62	2.2	0.21	4.2	0.769	43.75
				50	20.797	34.622	95.3	7.964	86.4	0.3	8.51	4.2	0.39	7.2	0.392	26.25
				80	18.351	34.625	92.6	7.906	77.5	0.6	2.28	6.2	0.64	12.4	0.168	72.61
				95	18.106	34.620	92.1	7.895	75.9	0.3	2.89	7.1	0.66	12.4	0.167	82.55
M4	121.9564	24.8179	244	3	24.368	34.541	95.9	8.039	95.8	0.3	3.41	0.1	0.10	1.6	0.866	9.95
				10	24.363	34.542	96.0	8.039	95.1	0.3	6.53	1.0	0.08	1.7	0.888	9.75
				25	24.215	34.547	96.1	8.019	95.5	0.3	8.92	1.6	0.15	2.9	0.906	17.14
				50	21.493	34.608	95.2	7.976	87.6	0.3	7.01	3.8	0.30	5.8	0.587	28.09
				100	19.882	34.640	94.5	7.942	83.5	0.1	4.67	8.9	0.54	7.5	0.301	31.22
