# Supplementary material for: Dominance of Sulfur-Oxidizing Bacteria, Thiomicrorhabdus, in the Waters Affected by a Shallow-Sea Hydrothermal Plume
Source: Biology (Basel). 2025 Jan 1;14(1):28. doi: 10.3390/biology14010028 (PMC11763282; doi:10.3390/biology14010028)
Supplement: Supplementary file 1 [file biology-14-00028-s001.zip › SuppTableS2_Ksd_16SSeqData.pdf]

Supplementary Table S2. The overall sequencing data of the 16S rRNA gene V3-V4 amplicons (16S rDNA) obtained from Guishan Islet during the period from 15 to 17 April 2019.

	Surface		Deep layer*	
	Reads	ASV	Reads	ASV
C3	400,376	1,652	315,959	1,650
H1	467,565	1,244	382,901	990
H4	451,023	1,951	394,226	2,009
M1	397,571	1,780	444,431	1,516
M2	524,270	1,779	438,913	1,699
M4	484,924	2,049	462,032	2,002

*Deep layer: the depth about 5 meters from the seafloor
