# Supplementary material for: Dominance of Sulfur-Oxidizing Bacteria, Thiomicrorhabdus, in the Waters Affected by a Shallow-Sea Hydrothermal Plume
Source: Biology (Basel). 2025 Jan 1;14(1):28. doi: 10.3390/biology14010028 (PMC11763282; doi:10.3390/biology14010028)
Supplement: Supplementary file 1 [file biology-14-00028-s001.zip › SuppTableS3_Ksd_RNAseqSum.pdf]

Supplementary Table S3. The overall sequencing data of the RNAseq obtained from the waters near the hydrothermal vent of Guishan Islet during the period from 15 to 17 April 2019.

Station	Depth (m)	Non-rRNA reads	Reads affiliated with *Thiomicrorhabdusg*	Reads affiliated with *Thiomicrorhabdusg* (%)
H1-Deep	15	6,783,567	2,026,637	29.8
H4-Surface	5	4,905,883	20,451	0.4
M1-Surface	5	7,720,058	39,884	0.5
M1-Deep	25	8,873,954	1,006,648	11.3
M4-Deep	25	3,562,117	139,462	3.9
